# Bluetongue in Captive Yaks

**DOI:** 10.3201/eid1404.071416

**Published:** 2008-04

**Authors:** Axel Mauroy, Hugues Guyot, Kris De Clercq, Dominique Cassart, Etienne Thiry, Claude Saegerman

**Affiliations:** * University of Liège, Liège, Belgium; † Veterinary and Agrochemical Research Centre, Brussels, Belgium

**Keywords:** vector-borne disease, bluetongue, yak, Bos grunniens, arbovirus, wildlife, case report, ruminant, letter

**To the Editor:** In August 2006, several northern European countries including Belgium reported cases of bluetongue (BT) (*1*). This noncontagious, arthropod-borne animal disease is caused by *Bluetongue virus* (BTV), genus *Orbivirus,* family *Reoviridae*. The genome of BTV consists of 10 segments of double-stranded RNA; 24 serotypes have been reported (*2*). Serotype 8 (BTV-8) was implicated in the emergence in Belgium (*3*). All ruminant species are thought to be susceptible to BT (*2*). We report laboratory-confirmed clinical cases of BT in yaks (*Bos grunniens grunniens*).

Yaks living in captivity in a Belgian animal park showed clinical signs of BT. A clinical examination performed on 1 yak showed loss of weight associated with a progressive weakness linked to anorexia, ulcerative and necrotic lesions on the muzzle with some crusts and mucopurulent nasal discharge, and udder erythema with papules and crusts. The tongue was severely swollen and cyanotic and protruded from the mouth (Figure). The animal was reluctant to move and was recumbent (possibly as a consequence of podal lesions linked to BT); it died 7 days after examination. Necropsies were performed on carcasses of this and another yak. The main lesions found were severe diffuse congestion of the lungs with edema and emphysema, acute hemorrhagic enteritis restricted to the ileum and jejunum, and petechial hemorrhages on the abomasums. No lesions characteristic of coronitis were noted.

**Figure:**
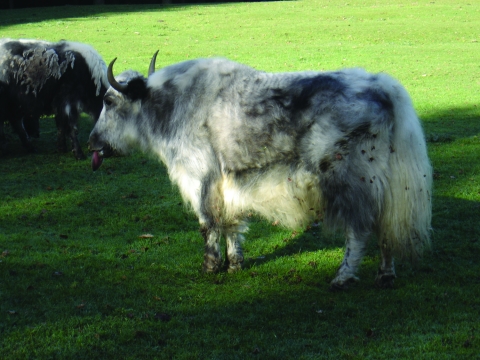
A captive yak infected with bluetongue virus. Tongue is swollen, cyanotic, and protruding from the mouth.

Samples of spleen and bone marrow were taken and prepared according to the method of Parsonson and McColl (*4*). A real-time reverse transcription quantitative–PCR (RT-qPCR) targeting BTV segment 5 (RT-qPCR_S5) was used to detect BTV RNA in tissues samples. Each test was performed in parallel with a RT-qPCR that amplifies β-actin mRNA as an internal control (RT-qPCR_ACT). Both assays were conducted according to Toussaint and others (*5*), with slight modifications. Briefly, total RNA was purified from 25 mg of tissue by Trizol extraction (Invitrogen, Carlsbad, CA, USA) and denatured by heating for 3 min at 95°C with 10% dimethylsulfoxide (Sigma-Aldrich, St. Louis, MO, USA). Reverse transcription reactions were conducted by using the Taqman reverse transcription reagents according to the manufacturer instructions (Applied Biosystems, Foster City, CA, USA). RT-qPCR reactions consisted of 1× concentrated Taqman fast universal PCR master mix (Applied Biosystems), 375 nM (β actin) or 500 nM (BTV) of each primer, 250 nM Taqman probe, and 5 μL cDNA. Cycling conditions were as follows: 1 cycle at 95°C for 20 s, followed by 45 cycles of 1 s at 95°C, and 20 s at 60°C. The specificity of the RT-qPCR used had been previously tested against prototype strains of genetically related viruses (9 strains of epizootic hemorrhagic disease virus and 9 strains of African horse sickness virus) (*5*). The RT-qPCR tests confirmed BTV viremia.

The yak species in its natural biotope is usually rarely exposed to competent *Culicoides* vectors. Antibodies against BTV have been found in many wild ruminants (*6*), and our results extend the number of ruminant species susceptible to BTV. In the northern European BT outbreak, lesions in cattle and sheep were mainly localized to the regions of the muzzle, mouth, and eye; clinical signs were not always obvious (*7*,*8*). As in cattle and sheep, clinical signs in yaks were observed on the muzzle, in the periocular region, and around and inside the mouth. These signs clearly reflected viral-induced endothelial damage triggering disseminated intravascular coagulation and a hemorrhagic diathesis commonly described in sheep and cattle (*2*). In our case, lesions depicted pronounced microvascular damage. According to the severity of the lesions and rates of illness and death observed, the yak, like sheep, appears to be highly susceptible to BTV.

In the epidemiology of BT in African countries, cattle and wild ruminant species such as antelopes play a role as asymptomatic reservoir hosts of the virus (*2*). Some wild ruminant species in captivity could also play this role in European countries affected by the recent BT outbreak. These cases could be of particular concern for all parks and zoos that gather numerous wild ruminants. Illness, reproductive failure, and deaths usually reported with BT (*9*) could generate substantial losses on these premises. Moreover, the source of BTV-8 in the northern European outbreak remains unclear, and the role of wild ruminant species has to be taken into account. In the future, European authorities should consider vaccination to prevent the spread of the disease in European member states (*10*). All premises with wild ruminants need to be involved in BT control and prophylaxis.
